# Analysis of cell‐specific peripheral blood biomarkers in severe allergic asthma identifies innate immune dysfunction

**DOI:** 10.1111/cea.14197

**Published:** 2022-08-02

**Authors:** Ben Nicholas, Jane Guo, Hyun‐Hee Lee, Alistair Bailey, Rene de Waal Malefyt, Milenko Cicmil, Ratko Djukanovic

**Affiliations:** ^1^ Division of Clinical and Experimental Sciences, University of Southampton Faculty of Medicine Southampton General Hospital Hampshire UK; ^2^ Oncology & Immunology Discovery Merck Research Laboratories Boston Massachusetts USA; ^3^ Cancer Sciences University of Southampton Faculty of Medicine, Southampton General Hospital Hampshire UK; ^4^ Merck Research Laboratories Palo Alto California USA

**Keywords:** allergy, asthma, biomarkers, peripheral, transcriptomic

**Key messages**

- Our study sought cell type‐specific gene signatures of severe allergic asthma in peripheral blood.
- We identified changes in monocytes and NK cells, but not T lymphocytes in severe asthmatics.
- Gene expression changes may serve as prognostic markers of severe asthma or as therapeutic targets.

To the editor,

Asthma is a common chronic airways disease with complex aetiology, having no single causative genetic trigger and with multiple factors appearing to contribute to disease pathogenesis.^1^ Thus, asthma can manifest in numerous different pathologies related to severity and the degree of allergy or response to therapeutic intervention. Currently, the principal clinical biomarkers for initial diagnosis and staging of disease severity include lung function and hyper‐responsiveness tests. Sometimes fractional exhaled nitric oxide (FeNO) is used as a surrogate for direct measurement of eosinophils. Improvement of clinical symptoms following therapeutic intervention and assessment of atopy and a history of wheeze are also useful.

Monitoring of the disease is generally through symptom assessment and therapeutic dose requirements combined with periodic lung function tests. Novel approaches, such as biologics targeting key disease pathways, have identified the utility of additional biomarkers such as IgE or sputum inflammatory cell counts as inclusion criteria for initiating treatment; however, in clinical practice, often the principal read‐outs remain the generalized clinical ones. No single test provides definitive evidence of asthma, its severity or therapeutic efficacy of drugs.

The aim of this study was to identify markers of cell type and asthma using gene expression profiles to identify patterns of disease in the periphery where they may most easily be examined. Recent reports have identified multiple disease phenotypes using unbiased clustering of genomic and proteomic data from airway samples.^2^ Likewise, mRNA signatures of severe asthma have been identified in whole blood.^3^ The importance of such studies may be confounded by known changes in inflammatory cell profiles in asthma, thus, missing changes in blood cell subtypes by defining the phenotypic clusters of subjects based on inflammatory cell predominance in biofluids. Our approach in the current study was to examine gene expression markers of cell type and asthma in four peripheral blood cell populations that have been a focus of interest for some time but have not yet been analysed in the level of detail as done in this study.

We recruited 11 severe allergic asthmatics on step 4 of BTS/Sign management (none on oral corticosteroids), and 10 healthy control subjects (see Table 1 for a summary of patient characteristics and online supplement for inclusion criteria: https://doi.org/10.6084/m9.figshare.20013536.v2). Participants signed written informed consent.

**TABLE 1:** Clinical characteristics for the healthy severe allergic asthmatic cohorts

	Healthy controls	Severe allergic asthmatics	*p* Value
*N*	10	11	‐
Gender (M/F)	3/7	3/8	‐
Age	42.5 (31–53)	43 (31–50)	.87
Atopy (yes)	0	11	‐
Total blood IgE	16.45 (10.7–27.8)	163.9 (71.4–315.0)	.0003***
Blood lymphocytes	1.9 (1.75–2.125)	1.9 (1.7–2.1)	.345
Blood eosinophils (%)	0.1 (0.1–0.2)	0.2 (0.1–0.3)	.212
Pre FEV1	3.11 (2.89–4.13)	2.58 (2.15–3.49)	.043*
FEV1% predicted (pre‐Salb)	111.4 (105.3–117.1)	100 (77.2–102.4)	.016*
FEV1 (post Salb)	3.19 (3.04–4.19)	3.00 (2.37–3.66)	.067
FEV1% predicted (post‐Salb)	114.1 (107.7–119.4)	100.6 (86.2–114.7)	.055
Sputum eosinophils (%)	0	1.69 (0–3.68)	.046*
Sputum neutrophils (%)	11.36 (7.1–15.7)	64.46 (21.9–74.2)	.006**
PBMC CD4^+^ (% of CD3^+^)	65.9 (53.5–71.2)	70.3 (58.5–78.5)	.251
PBMC CD8^+^ (% of CD3^+^)	26.7 (18.6–33.0)	23.9 (16.8–26.5)	.340
PBMC monocytes (% of CD45^+^)	11.7 (10.2–16.4)	12.8 (8.7–14.0)	.878
PBMC NK cells (% of Cd45^+^)	11.1 (8.8–19.5)	10.6 (7.4–11.7)	.314

*Note*: Mann Whitney tests, *p < 0.05, **p < 0.01, ***p < 0.001. Data shown are medians (interquartile range). Salb: salbutamol.

Fresh blood was drawn and PBMCs were isolated from buffy coats. Flow cytometry determined no significant differences in the relative proportions of four immune cell populations, monocytes, NK cells, CD4^+^ and CD8^+^ T cells in the peripheral blood of healthy and severe asthmatic subjects (Table 1).

Four cell populations were sequentially enriched from 4 × 10^7^ PBMCs from each subject using magnetic beads conjugated to monoclonal antibodies targeting different cell surface markers, namely CD14 (monocytes), CD56 (NK/NK‐T cells), CD8 (CD8^+^ T cells) and CD4 (CD4^+^ T cells) in that order. This yielded populations highly enriched (>99%) for each selective marker.

Quantification of gene expression used isolated RNA from the four enriched cell populations, analysed by a 770 gene nCounter pancancer immune profiling array (NanoString Technologies).

Unbiased principal component analysis (PCA) of the purified cell gene expression profiles robustly separated the four target cell populations (Figure 1A). We found no evidence of segregation (clustering) between asthma and health in these cell populations. The genes primarily driving cluster separation of each cell type, and as such indicative of cell‐specific biomarkers, confirm the purity of our immune blood cell populations and add to the canon of knowledge on markers of these cell types (see online data supplement for cell‐specific gene lists: https://doi.org/10.6084/m9.figshare.19329068).

**FIGURE 1:**
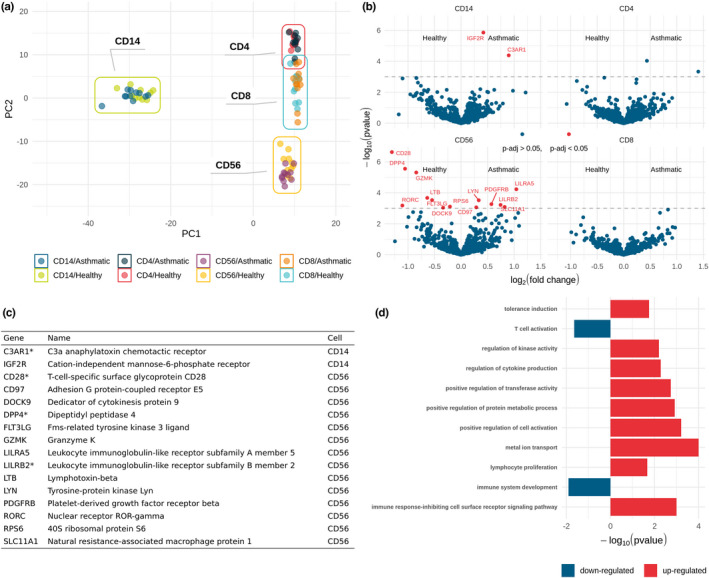
Differential gene expression analysis in RNA transcripts in severe asthma. (A) PCA analysis of gene counts from all four cell types indicate clusters corresponding to each cell type. (B) Volcano plots indicating genes significantly up‐ or downregulated in severe asthma in each cell type (*p*‐adjusted <0.05, points in red above the dashed line). (C) Table of significantly upregulated genes corresponding to red points in volcano plots. Genes previously observed as altered in peripheral blood in a severe asthma cohort^3^ are highlighted with an asterisk. (D) Summary of GO terms associated with differential gene expression in NK cells in asthma.

For example, a major component of the monocyte cluster was CD14, which was to be expected since it was the target isolation molecule, but also included 425 other genes contributing to this cluster, inclusive of the well‐characterized monocyte‐specific proteins such as CD68, CD163, L‐selectin and S100A12.

T cells exhibited a well‐defined cluster; however, a small degree of overlap between the CD4^+^ and CD8^+^ T‐cell clusters, reflects the phenotypic similarity between these two cell types.

Similarly, our top‐ranked genes contributing to the NK cell cluster were well‐known phenotypic markers of NK cells, inclusive of KLRD1, NCAM‐1 and EOMES. Clusters were driven primarily by cell type.

We then identified individual differentially expressed genes (DEGs) in asthma, adjusting for multiple tests for each cell type as appropriate. Volcano plots (Figure 1B,C) indicated two genes upregulated in asthmatic monocytes compared to healthy controls (IGF2R and C3AR1). IGF2R is known to be upregulated in monocytes during differentiation into macrophages,^4^ but changes in IGF2R expression by monocytes in asthma have not previously been observed. C3AR1 has been previously identified as a susceptibility gene for bronchial asthma.^5^ Although C3AR1 has been previously associated with severe asthma in whole blood,^3^ expression was not localized to monocytes.

We found six upregulated (LILRA5, LILRB2 and SLC11A1, PDGFRB, LYN, CD97) and eight downregulated (LTB, RORC, GZMK, DPP4, RPS6, FLT3LG, DOCK9 and CD28) genes in CD56^+^ cells from asthmatics (Figure 1B,C). LILRB2 has been previously associated with severe asthma and CD28 and DPP4 were both associated with health in peripheral blood cells in a previous study.^3^ Our data confirms these findings, adds further to the list of DEGs, and also indicates the cellular source of these gene expression patterns.

Several of these upregulated genes are associated with immune cell regulation. For example, the leukocyte immunoglobulin‐like immunoregulatory checkpoint receptors LILRA5 and LILRB2 have both positive and negative regulatory properties, whereas LYN‐dependent signalling is primarily a negative regulator of innate and adaptive immune responses.^6^ Additionally, the PDGF pathway has a well described local airways role in asthma pathogenesis and airway remodelling. Increased systemic levels of PDGF ligands in the serum of asthmatics have been previously observed; however, it has remained uncertain whether PDGF is a systemic biomarker of asthma.^7^ Our study suggests that PDGF‐receptor expression may provide evidence of systemic response to abnormal PDGF expression in asthma.

SLC11A1, another upregulated gene in asthmatic NK cells, is involved in pathogen resistance and plays a role in divalent metal ion transport, promoting anti‐microbial pathogen responses. This protein is upregulated in local innate lymphocytic cell subtypes in asthma, augmenting their activation and biasing towards a Th1 rather than Th2 response. Abnormal SLC11A1 expression has previously been identified in asthmatic sputum cells,^8^ attributed to localized bacterial exposure. Systemic changes in expression of SLC11A1 may reflect the disease severity and the consequent neutrophilic phenotype of our severe asthma cohort. However, the existence of SLC11A1 upregulation in peripheral blood is suggestive of widespread systemic innate cell dysfunction.

Conversely, several genes downregulated in CD56^+^ cells of asthmatics (LTB, RORC, GZMK, DPP4, RPS6, FLT3LG, DOCK9 and CD28) are associated with proinflammatory, functional, cell or activation responses in NK cells. For example, lymphotoxin beta is a proinflammatory TNF‐α superfamily member, granzyme K is a serine protease and component of cytotoxic granules in NK cells, and CD28 is a co‐stimulatory molecule for NK cells. Interestingly, DPP4, which plays a role in NK cell activation,^9^ is one of the ligands of IGF2R, which is elevated in monocytes and may be suggestive of common systemic pathway alterations in peripheral innate cells.

Pathway analysis, using the differentially expressed genes in the NK cell‐enriched population (Figure 1D), confirmed that severe allergic asthma was associated with upregulation of pathways involved in metabolism, divalent metal ion transport, immune response inhibition and immune regulation/tolerance, and with downregulation of T‐cell activation and immune system development.

Overall, these data point to induction of tolerance mechanisms, at least in the circulating cells that we studied. As a consequence, the observed NK cell dysfunction could impair the adaptive immune response to microbes, possibly reflecting exhaustion or generalized impairment of these cells. However, at the same time, the data show enhanced anti‐microbial responses in the absence of recent exacerbation that are, to a large extent, induced by infection. Although NK and NK‐like cells have been examined in asthma, no fixed role for them has yet been assigned. Our study suggests that further investigation of these cells is warranted.

Additionally, monocytes in asthma showed increases in gene expression associated with differentiation and chemotaxis. Thus, we found innate but not adaptive cell phenotypic changes in peripheral blood cells from severe asthmatics.

The limitations of this study include a relatively small cohort and comparison of healthy with severe allergic asthmatics, meaning that some observed differences could be due to atopy or other clinical characteristics rather than asthma per se. We also did not assess the most severe asthmatics who require oral corticosteroids. Finally, the lack of an independent validation group means that any potential biomarkers would have to be investigated in future studies.

In summary, the findings presented herein may help to identify new therapeutic avenues for severe asthma arising from innate immunity and also may serve to identify new gene candidates as useful cell type‐specific biomarkers. Given the increasing interest in innate immunity and its role in anti‐microbial responses, these results suggest the value of further study of gene expression changes at times of acute asthma exacerbations.

## AUTHOR CONTRIBUTIONS

Conceptualization: Ben Nicholas, Jane Guo, Hyun‐Hee Lee, Rene de Waal Malefyt, Milenko Cicmil and Ratko Djukanovic. Investigation: Ben Nicholas, Jane Guo and Hyun‐Hee Lee. Analysis: Ben Nicholas and Alistair Bailey. Writing: Ben Nicholas, Alistair Bailey, Ratko Djukanovic.

## CONFLICT OF INTEREST

The authors have declared no competing interest.

## Data Availability

All data produced in the present work are contained in the manuscript.
